# Establishing gaze markers of perceptual load during multi-target visual search

**DOI:** 10.1186/s41235-023-00498-7

**Published:** 2023-08-31

**Authors:** Anthony M. Harris, Joshua O. Eayrs, Nilli Lavie

**Affiliations:** 1 Institute of Cognitive Neuroscience, University College London, London, UK; 2 Queensland Brain Institute, The University of Queensland, Brisbane, Australia; 3 Department of Experimental Psychology, Ghent University, Ghent, Belgium

**Keywords:** Attention, Perceptual load, Visual search, Eye movements, Cross-modal processing

## Abstract

Highly-automated technologies are increasingly incorporated into existing systems, for instance in advanced car models. Although highly automated modes permit non-driving activities (e.g. internet browsing), drivers are expected to reassume control upon a ‘take over’ signal from the automation. To assess a person’s readiness for takeover, non-invasive eye tracking can indicate their attentive state based on properties of their gaze. Perceptual load is a well-established determinant of attention and perception, however, the effects of perceptual load on a person’s ability to respond to a takeover signal and the related gaze indicators are not yet known. Here we examined how load-induced attentional state affects detection of a takeover-signal proxy, as well as the gaze properties that change with attentional state, in an ongoing task with no overt behaviour beyond eye movements (responding by lingering the gaze). Participants performed a multi-target visual search of either low perceptual load (shape targets) or high perceptual load (targets were two separate conjunctions of colour and shape), while also detecting occasional auditory tones (the proxy takeover signal). Across two experiments, we found that high perceptual load was associated with poorer search performance, slower detection of cross-modal stimuli, and longer fixation durations, while saccade amplitude did not consistently change with load. Using machine learning, we were able to predict the load condition from fixation duration alone. These results suggest monitoring fixation duration may be useful in the design of systems to track users’ attentional states and predict impaired user responses to stimuli outside of the focus of attention.

**Supplementary Information:**

The online version contains supplementary material available at 10.1186/s41235-023-00498-7.

## Introduction

Cognitive neuroscience research has established that a major determinant of attentional engagement in a task is the level of perceptual load it involves. Conditions of high perceptual load (for example, tasks with an increased number of stimuli, or a search task with more similar target and non-target items) result in reduced perception of signals outside the attended task, leading to phenomena of load-induced ‘inattentional blindness’ (e.g. Cartwright-Finch & Lavie, 2007) and ‘inattentional deafness’ (McDonald & Lavie, 2011; Raveh & Lavie, 2015). Impairments of this type could be detrimental if they affect the user’s ability to perceive a warning signal in a safety critical scenario. As such, it is important to develop measures that allow estimation of the likelihood that a user will perceive and respond to a warning signal in a timely manner.

A ‘user case’ that is becoming increasingly pervasive comes from the development towards fully automated vehicles (Level 5 of driving automation; SAE, 2021, that are often termed the ‘driverless car’). Level 3 of automation has recently been approved for commercial use in some areas (e.g. Germany and Nevada, USA). Level 3 automation differs from previous driver-assist systems in providing an ‘autonomous’ driving mode that frees the driver from any driving-related activity, including watching the road. This will eventually allow the driver to engage in any non-driving task of their choice (e.g. internet browsing, online shopping etc.)^1^. However, upon the presentation of a warning signal that indicates that the AI control of the car will imminently cease (e.g. in case the road conditions cannot be handled by the car’s computer vision system) the driver must swiftly take over control of the driving. This warning signal is termed the Take Over Request (TOR), and an important topic under current investigation concerns establishing the determinants of drivers’ ability to promptly respond to the TOR and take over control of the car. Since perceptual load is a major determinant of the ability to perceive and respond to stimuli outside the current focus of attention, it is important to establish a non-intrusive method for detecting a user’s current level of perceptual load. Eye tracking is one promising avenue for such detection. Indeed, pupil dilation has been established as a marker of perceptual load (e.g. Porter et al. 2010; Oliva, 2019); however, due to the sensitivity to light and difficulty in accurate registration of the pupil area when the eyes are free to move (e.g. Mathur et al., 2013), such a method is hard to implement outside the laboratory. Tracking of gaze fixations and movement patterns may provide a better applied solution.

However, the attention tasks previously used to establish the effects of perceptual load have not typically permitted free eye movements, instead using very brief display presentations and having participants detect or search for a single target item. In contrast, the real-life tasks that a driver may wish to engage in during the autonomous driving mode may involve longer presentations, free eye movements and more than one ‘target’. For example, a driver may choose to engage in internet shopping during the automated driving mode, and this may involve search for multiple potential target products, and multiple candidates for each target (e.g. a search for a new compact-sized car with a low level of emissions would involve several ‘short-listed’ car models to compare). In addition, some browsing may involve greater attentional requirements (higher perceptual load) with no differences in the visual images inspected, simply due to greater processing requirements for these images. For example, searching for a combination of features (an L shaped sofa in crimson colour, with the corner on the left) involves greater processing requirements than search for a single feature (any crimson sofa). Finally, the driver may not respond manually to the search items of interest, but rather scrutinise them with greater attentional engagement (i.e. gaze longer at the item) before moving on to the next potential target. In contrast, previous perceptual load tasks, like most laboratory tasks, typically required manual response (e.g. a key press or a mouse click) to indicate a target was found. In either scenario, a warning signal such as the TOR may be presented, and if the task consumed full driver attention, the driver may fail to promptly detect and respond to the TOR.

In the present research, we designed a multi-target gaze-based visual search task that mimics all these real-world task features. In this task, people only needed to scan the displays and linger their gaze on the search targets for a predefined period. Additionally, they were required to stop their search and respond promptly to an auditory tone (serving as our takeover proxy). We measured the impact of perceptual load on the responses to the TOR proxy as well as on the gaze properties in the search task. Below we briefly review the relevant previous research.

## The impact of perceptual load on detection outside the focus of attention

Perceptual load theory proposes that perceptual resources are finite and are always allocated in full, so that unused resources spill over to process items outside the focus of attention (e.g. Lavie, 1995, 2005). The greater the perceptual load of the task, the fewer resources remain for the processing of stimuli outside of the central task. For example, when participants complete a demanding central task (e.g. judging whether the horizontal or vertical line of a cross is slightly longer), they are less likely to detect the appearance of a salient object in the periphery than if they complete a less demanding task (e.g. judging whether the horizontal or vertical line was blue), (Cartwright-Finch & Lavie, 2007). Similar effects have been observed when participants complete a visual search in which targets are highly similar to distractors, for example an ‘X ‘ or ‘N’ among other angular letters (high load), versus a visual search in which targets and distractors are highly dissimilar, such as an ‘X’ or ‘N’ among small ‘o’ stimuli (low load), (Macdonald & Lavie, 2008).

Further work has demonstrated that the effects of perceptual load apply cross-modally. When performing a high load visual task, participants are less likely to detect a secondary auditory stimulus than when performing a low-load visual task (Macdonald & Lavie, 2011; Molloy et al., 2015; Murphy & Greene, 2015; Raveh & Lavie, 2015). Raveh and Lavie (2015) had participants perform the same letter search as described above (Lavie & Cox, 1997; Macdonald & Lavie, 2008), but presented auditory detection stimuli during the search on a subset of trials (17% in Experiments 1 & 2, 50% in Experiments 3 & 4). In each experiment, they found that participants had significantly higher sensitivity (d’) for detecting auditory tones presented on low-load trials, than for the same tones presented on high-load trials. Murphy and Dalton (2016) established similar effects in the domain of somatosensation, with high visual perceptual load impairing detection sensitivity for tactile stimuli. These results demonstrate that attentional limitations brought about by performing a taxing visual task impair stimulus processing in other sensory modalities.

This line of research has also been extended to the domain of driving. Murphy and Greene (2015) had participants perform a simulated driving task, during which they manipulated perceptual load in two ways. In Experiment 1, participants were required to judge whether their car would fit between cars parked to either side. These judgements were either obvious (cars very close or very far apart; low load) or difficult (cars slightly too close or just far enough apart; high load). In Experiment 2, they performed a search for a red Mercedes parked either among silver cars (low load) or among other red cars (high load). In both experiments, on a critical trial the participants were presented with an unexpected stimulus (e.g. a person beside the road, or the sound of screeching tyres), and immediately following the trial were probed as to whether they perceived the unexpected stimulus. Both visual and auditory stimuli were far more likely to be missed when participants were performing a high-load driving task, even when the unexpected stimuli were driving relevant. Together, the studies reviewed here suggest it is likely that performing a high-load task in the car will have a detrimental impact on driver responses to a TOR.

The tasks described above have produced robust effects of perceptual load in numerous studies (e.g. Beck & Lavie, 2005; Carmel et al., 2007; Cartwright-Finch & Lavie, 2007; Forster & Lavie, 2007, 2008a, 2008b, 2009, 2016; Gupta et al., 2016; Lavie & Cox, 1997; Macdonald & Lavie, 2008, 2011; Raveh & Lavie, 2015; Remington et al., 2009). Another manipulation of perceptual load that has been well studied and has the added benefit of matched stimuli between load conditions is the feature versus conjunction task (e.g. Bahrami et al., 2008; Carmel et al., 2006, 2011; Molloy et al., 2019; Murphy & Greene, 2017; Schwartz et al., 2005). In this task, participants are presented with the same search stimuli (e.g. a rapid stream of upright and inverted cross stimuli of different colours) in high and low perceptual load conditions), and load is manipulated through whether the target definition is feature-based (low load, e.g. any red cross) or a conjunction of features (high load, e.g. targets are upright green crosses and inverted yellow crosses). The same manipulation has been employed spatially, with search arrays of different shapes presented simultaneously (Molloy et al., 2019). As in the experiments described earlier, both visual and auditory detection sensitivity of a task-unrelated item was decreased in the high-load condition, showing perceptual load affects detection performance under this distinct manipulation of load (see also, Murphy & Greene, 2017).

## Fixation duration and perceptual load

To the best of our knowledge, apart from the known effects of perceptual load on pupil dilation patterns (e.g. Oliva, 2019; Porter et al., 2010) other gaze correlates of perceptual load remain to be investigated. Nevertheless, some visual search studies are suggestive of additional gaze metrics that may be sensitive to the effects of perceptual load.

Several studies have reported that fixation durations tend to be longer when search is more difficult (e.g. Hooge & Erkelens, 1996; Horstmann et al., 2016; Vlaskamp & Hooge, 2006; Zelinsky & Sheinberg, 1997). For example, Hooge and Erkelens (1996) asked participants to search for a target circle presented with six Landolt-C non-targets. Search difficulty was manipulated by reducing the size of the gap in the distractor Landolt-C stimuli. They found that fixation durations were increased when search was more difficult. Similarly, Vlaskamp and Hooge (2006) manipulated the distance between search items and irrelevant non-search items so as to adjust the degree of crowding the stimuli were subject to. They found that increased crowding produced a corresponding increase in fixation duration. Horstmann and colleagues (2016) had participants search for a target emotional face among neutral non-target faces. They found that when participants knew the target would be similar (a face showing a slight frown) to the neutral distractors, non-target fixations were extended relative to when the target was dissimilar (a face showing a strong grimace with teeth visible) to the neutral distractors.

Some of the manipulations described above involved a change in the retinal acuity of the stimuli that has co-varied with the conditions of task difficulty (e.g. reducing the gap size of a Landolt-C and increasing crowding will both reduce the retinal acuity of the stimulus presented). Given the drop in retinal acuity from the fovea to the retinal periphery, such stimuli clearly require foveation to be reliably resolved. It is thus not clear to what extent these factors were responsible for the longer durations of fixations, instead of or in addition to, the impact of perceptual load.

Some eye-tracking search studies have compared gaze properties under feature versus conjunction search. It is well established that conjunction search places a higher load on attention than feature search and this can result in detection failures and reduced primary visual cortex response to unattended stimuli (e.g. Bahrami et al., 2008; Carmel et al., 2011; Jacoby et al., 2012; Schwartz et al., 2005). Zelinsky and Sheinberg (1997) had participants search for either feature singleton targets (e.g. a red bar among green bars) or conjunction targets (e.g. a red horizontal bar among red vertical and green horizontal bars). They found no difference in fixation duration between the conditions. The majority of studies employing this manipulation, however, have tended to show longer fixations under conjunction search than feature search (e.g. Pomplun et al., 2013; Porter et al., 2010, Scialfa & Joffe, 1997, 1998; but see Tagu & Kristjánsson, 2022 for an example of conjunction search resulting in shorter fixations than feature search). These results give a promising indication that increased perceptual load may be associated with increased fixation duration. However, past studies have all incorporated stimulus differences between feature and conjunction search that make it difficult to be certain that differences in fixation duration are due to perceptual load and not due to differences in properties known to affect fixation durations such as local feature contrast (Nuthmann, 2017) or crowding and clutter (Nuthmann, 2017; Vlaskamp & Hooge, 2006). To address this question, it is important that studies are performed with stimuli that are as matched as possible between the perceptual load conditions. Further, it is critical to incorporate an independent measure of perceptual load, such as evidence of a decrement in processing task-irrelevant stimuli (e.g. Cartwright-Finch & Lavie, 2007; Macdonald & Lavie, 2008; see Lavie et al., 2014, for review). Indeed, studies showing that perceptual load in vision can reduce detection of stimuli in the auditory modality, as well as reduce the associated neural signals (e.g. Macdonald & Lavie, 2011; Molloy et al., 2015; Raveh & Lavie, 2015), are particularly immune to any local visual sensory interactions as an explanation for the effects of perceptual load. As mentioned earlier, gaze behaviour is likely to be sensitive to visual sensory factors that lead to reduced retinal acuity, which can be compensated for by longer foveation. As such, we deemed it important in the present study to assess the effects of perceptual load on gaze behaviour in a design that validates the perceptual load manipulation by assessing the cross-modal impact of visual perceptual load on auditory processing.

In addition, previous visual search studies, including those examining perceptual load and those that show gaze correlates of visual search, have typically involved search for only one target. While clearly informative in a number of ways, these designs are limited in the extent to which they can be generalised to real-world tasks that are typically ongoing after a potential target is found. This is particularly important in an applied scenario, where gaze behaviour may change over time as a task continues. For instance, Mills et al. (2011) observed across four different natural scene viewing conditions (search for a hidden letter, viewing for pleasantness rating, viewing for later recall, and free viewing), that fixation durations increased across the several seconds of trial performance. In contrast, saccade amplitudes increased across the first ~ 1.5 s and then stabilised. Thus, whether and how fixation and saccade behaviour change across time in different load conditions is an important question for the estimation of attentional states in applied scenarios. To answer this question requires tracking of the gaze over extended periods of task performance, such as search for multiple targets under conditions of low or high perceptual load.

## The present study

We designed a task that incorporates principles of real-world search and attentive eye scanning behaviour, such as internet shopping. In these situations, gaze markers may be particularly sensitive to detecting when the task involves a high level of perceptual load that has a detrimental effect on responses to an auditory signal (our TOR signal proxy). Participants were presented with a multi-target visual search task in which an auditory tone was presented during some of the search displays, and on those trials, participants were required to abort the visual search and respond as fast as possible to the tone. This aspect of the task mimics real-world scenarios in which the operator needs to respond to a warning signal by aborting their current task and switching to another task. For example, in the case of highly automated driving, a TOR could indicate the need for a human to cease their non-driving tasks and resume driving. As another example, in the case of surgery, safety–critical monitor signals may indicate the need for immediate attention to a separate problem (e.g. a sudden reduction in blood pressure). In addition, any impact of load on the perception of, and response to, a task-irrelevant stimulus, especially one that is presented in a different sensory modality, validates the Load Theory proposal that these effects of load reflect the greater engagement of attentional resources. This was particularly important to our purpose of studying gaze markers of load that clearly indicate a greater level of attention engagement, rather than, for example, a compensatory strategy to mitigate the effects of reduced visual acuity.

**Fig. 1:**
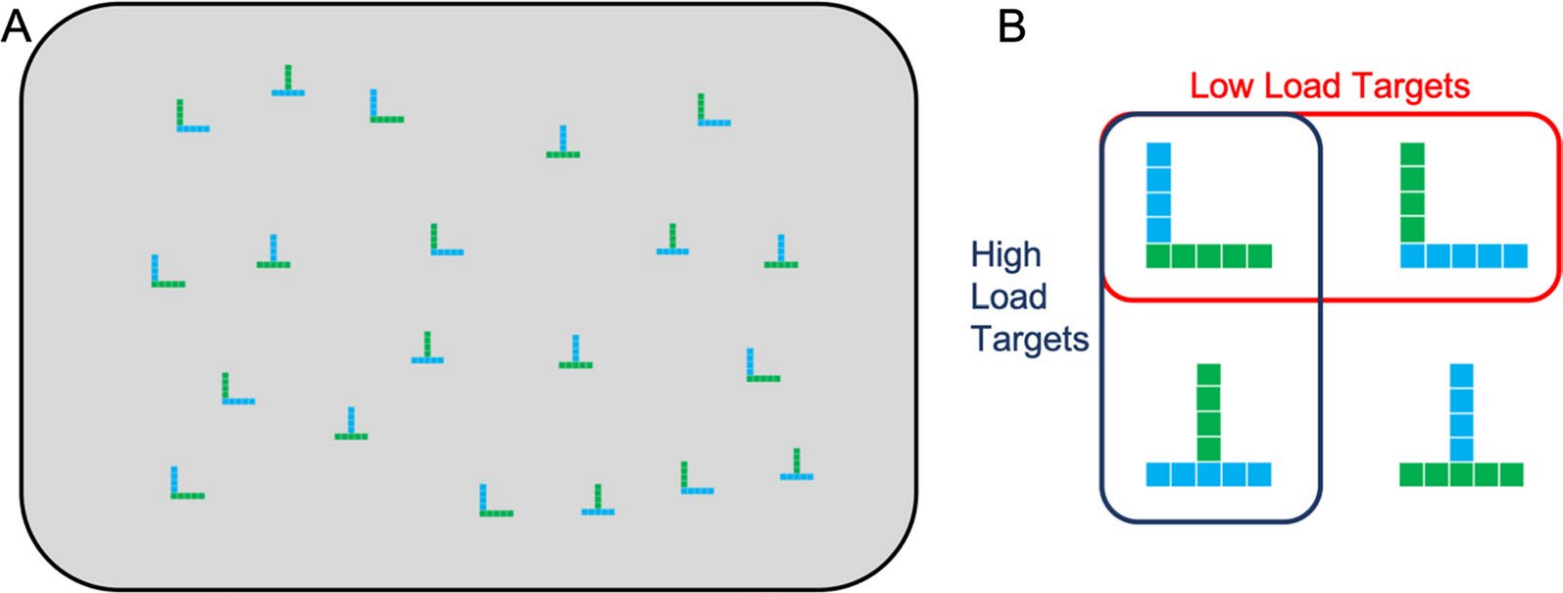
Example stimuli. **A** Example search display. **B** Stimuli used in each display. Low-load targets were any L shape. High-load targets were Ls with green horizontal portions and blue vertical portions, and upside-down Ts with blue horizontal portions and green vertical portions

We also deemed it important that the displays do not differ under perceptual load, since otherwise the perceptual complexity of the display itself can be used as a marker of perceptual load (Nagle & Lavie, 2020). Therefore, our search task involved T and L shapes, each shape composed of different coloured horizontal and vertical parts, and perceptual load was manipulated by whether the target was defined by shape (all Ls) or by different combinations of colour and orientation within each of the shapes (Ls with green horizontal portions and blue vertical portions, and Ts with blue horizontal portions and green vertical portions, see Fig. 1). Participants also searched under time pressure rather than performing an exhaustive search. In this way, the task not only involved varied perceptual load for the same displays, but also required a high level of focus in the high-load conditions, while precluding a simplifying run-based strategy (Kristjansson et al., 2018).

To further mimic real-world search situations in which users’ hands are not free to respond to targets (such as when a driver’s hands must be kept on the wheel, when a shopper is simply perusing items online without selecting any, or when a surgeon must use their hands for the operation), we asked the participants to indicate a target by lingering their fixation on it rather than responding manually (Jóhannesson et al., 2016; Kamienkowski et al., 2012). As participants performed the search, their gaze behaviour was measured with a video-based eye-tracker. We compared tone detection rates and response times, search performance, as well as fixation durations and saccade amplitudes between high- and low-load search. If properties of the gaze such as fixation duration on non-targets or saccadic amplitude distinguish between high versus low perceptual load search conditions, such measures may be used to predict the user’s readiness to detect and respond to safety–critical signals such as a TOR.

## Experiment 1

### Methods

#### Participants

Twenty participants completed this experiment (14 females, mean age = 23.35 years, *SD* = 2.98 years). Participants were recruited from the UCL Institute of Cognitive Neuroscience subject mailing list and were compensated for their time at a rate of £7.50 per hour. Participants had normal hearing and normal vision (no glasses or contact lenses) with no self-reported colour vision deficiency or astigmatism. The experiment was approved by the UCL Research Ethics Committee.

#### Apparatus and stimuli

The experiment was programmed with the psychtoolbox extension (Brainard, 1997; Kleiner et al., 2007) for Matlab, under the Windows 10 operating system. Visual stimuli were presented on a Dell S2417DG 23.8-inch LED monitor with a display resolution of 1920 × 1080 pixels and a 60 Hz refresh rate, placed 66.5 cm from the participant. Manual responses were collected with a standard USB keyboard. Auditory stimuli were presented via a pair of Sennheiser HD 598 headphones connected to an RME Fireface UC sound card. Eye movements were recorded with an Eyelink 1000 Plus video-based infrared eye-tracker, sampling monocularly from the right eye at 500 Hz. Participants rested their chin in a chinrest with their forehead touching a padded forehead bar attached to the chinrest. The experiment was run in a dimly lit room.

Search stimuli were presented on a grey background. Prior to the appearance of the search display, a fixation display was presented which consisted of the grey background with a small black fixation cross (0.25° × 0.25°). Upon appearance of the search display the fixation cross disappeared. The search stimuli were Ls and upside down Ts, each roughly 0.7° × 0.7°, and made up of five small squares of the same colour (blue or green) in the horizontal portion, and four small squares of the other colour in the vertical portion of the shape (Fig. 1). There were 20 stimuli in each search display: 5 Ls with green horizontal and blue vertical portions, 5 Ls with blue horizontal and green vertical portions, 5 Ts with green horizontal and blue vertical portions, and 5 Ts with blue horizontal and green vertical portions.

Visual search stimuli were presented within a central 32° × 24° region of the display so as to conform to the accurate trackable range of the eye-tracker. On each trial, stimuli were allocated randomly to positions of a 10 × 7 grid, with grid positions separated by roughly 3°. Each stimulus then had its vertical and horizontal position jittered by up to 0.5° to reduce the collinearity of stimuli in the same row or column. On trials in which tones were presented, the tone was a clearly audible 400 Hz pure tone of 38.6 dB presented for 50 ms.

#### Procedure

Informed consent was obtained from the participants prior to the experiment. Participants were then given both written and verbal instructions on how to perform the task. Prior to beginning the task, participants were exposed to the tone at the same volume and duration as was used in the task. They were asked whether they could perceive the tone, and the veracity of their report was tested by presenting the tone at two random times and asking the participant to indicate when they heard the tone. All participants were easily able to perceive the tone, and this was confirmed by ensuring the participants responded to tones during the practice blocks of the task.

In the experiment, participants performed a visual search task in which they searched for targets of two predefined types that differed between two load conditions. In the low-load condition, the targets were the two L shapes, so search could be completed on the basis of shape alone. In the high-load condition, targets were defined by conjunctions of shape and colour; they were the L with green horizontal and blue vertical sections, and the upside-down T with blue horizontal and green vertical sections. There were 10 targets per display, 5 of each type.

Load conditions were presented in blocks of 36 trials each. Each block began with an instruction screen, informing participants which stimuli were the targets, and which were the non-targets for that block. Participants were encouraged to look at the example stimuli on the instruction screen until they felt confident that they would remember which were their targets. They then began the block with a keypress. Each block began with the eye-tracker’s standard 9-point calibration procedure. Once a suitable calibration had been achieved, resulting in a validation error of less than 1° at all points (*M* = 0.33°, SD = 0.21°), the task began.

Each trial began with a fixation display. The search display was presented once participants had fixated within 0.5° of the centre of the central fixation cross for 500 ms, unbroken by blinks or eye movements. If 3 s passed without this criterion being reached, participants were calibrated anew, and the trial began again with the fixation control procedure.

Once the search array appeared, participants were required to find as many targets as possible within the 7-s duration of the trial. This duration was selected so that participants would be unlikely to find all of the targets (indeed, no participant found all the targets on any trial), thus allowing us to compare the number of targets detected between the load conditions. Participants moved their eyes around the display and, once a target was found, were required to fixate within 0.95° of the target for 500 ms, unbroken by blinks or eye movements, upon which time the target would disappear from the display and the participant could continue their search to find more targets. Target fixations were required to be 500 ms as this duration is significantly longer than the average duration of a fixation (roughly 250 ms), ensuring targets would not disappear if participants simply moved their eyes through the display randomly without performing the search (this was confirmed during pilot testing). Extended fixation upon distractors did not cause them to disappear. At the end of the trial, the search array disappeared, and the next trial would begin with the fixation control.

On 50% of the displays, a tone was presented via the headphones. Each tone appeared at one of 18 equally spaced times between 2 and 5 s (i.e. at either, 2 s, 2.18 s, 2.35 s, and so on) on randomly selected search displays in each block. Upon hearing the tone, participants were required to abort their search and press the spacebar as quickly as possible. Participants were instructed to rest their dominant hand on the spacebar throughout the task. The trial ended as soon as the spacebar was pressed, or after 7 s from the beginning of the trial if no response was made.

A self-paced break occurred at the end of each block. Participants were given no feedback about their performance on either the search or tone tasks.

Participants first completed two practice blocks of 6 trials each, one block of each load level, followed by 8 experiment blocks of 36 trials each, presented in a counterbalanced order, either LHHLHLLH or HLLHLHHL, where L indicates a low-load block and H indicates a high-load block.

#### Eye-tracking parameters

For the eye-tracking analyses, saccades were defined with standard parameters (velocity greater than 30°/s or acceleration greater than 8000°/s^2^). Fixations were defined as any time in which the pupil was visible, and a saccade was not being made. Blinks were removed from the analysis along with their associated pre-blink and post-blink saccades (caused by rapid occlusion of the pupil being interpreted by the eye-tracker as a saccade).

#### Machine learning analysis

To examine whether fixation durations and saccade amplitudes could be used to predict load conditions, we performed a Support Vector Machine (SVM) classification analysis, classifying trials as either high or low load based on their gaze properties. Performance of the model was assessed on a per-participant basis via tenfold cross-validation. That is, the model was trained on 90% of the data and tested on the 10% of data that was left out, and this procedure was repeated until all the data had been used in the testing set. In every test, it was ensured that both load conditions contained an equal number of data points by selecting a random number of data points from the larger set equal to the number of data points in the smaller set. As this analysis is intended as a proof of concept for the predictive value of the variables involved, rather than an attempt to build an application-ready model, we left aside any attempts at iterative model tuning. We report the average accuracy, precision, recall (sensitivity), and F1 values (an overall metric that combines precision and recall) obtained using the default MATLAB model parameters. Only accuracy was submitted to statistical analysis, as we were interested in assessing whether perceptual load can be predicted from our variables, not in the quality of these specific model implementations.

### Results and discussion

#### Search task

High perceptual load was associated with a smaller number of targets found (*M* = 5.33) compared to low load (*M* = 7.14), *t*(19) = 27.18, *p* < 0.001, *Cohen’s D* = 4.10, and a greater number of non-target fixations (*M* = 6.11 vs. *M* = 3.35 in the low-load condition), *t*(19) = 24.41, *p* < 0.001, *Cohen’s D* = 4.25. Average values for each of the assessed gaze properties are presented in Table 1. Fixation duration was assessed for non-target items only, since the task required that target selection was indicated by a prolonged fixation of 500 ms. Non-target fixation durations were significantly longer under high load than under low load (Table 1), *t*(19) = 11.73, *p* < 0.001, *Cohen’s D* = 2.83. There were more false positives (distractors fixated for longer than 500 ms) in the high load than in the low-load condition; however, after excluding these fixations, the difference in fixation duration between high- and low-load search remained, *t*(19) = 11.16, *p* < 0.001, *Cohen’s D* = 2.42. Saccade amplitude did not differ between the load conditions (Table 1), *t*(19) = 1.11, *p* = 0.283, *Cohen’s D* = 0.25.

**Table 1:** Means and standard deviations (in parentheses) for the gaze metrics as a function of perceptual load in Experiment 1

	Fixation duration (ms)	Fixation duration (ms) excl. False Pos	False positives (# per trial)	Saccade amplitude (degree)
Low load	222 (28)	191 (21)	0.16 (0.08)	4.93 (0.33)
High load	324 (43)	238 (18)	0.96 (0.31)	4.84 (0.36)

#### SVM predictions

An SVM classifier trained on participants’ average fixation durations from each trial was able to classify the load condition of each trial well above the chance level of 0.5 (Table 2). This pattern of results persisted when classifying the load condition associated with single fixations. In addition, although there was no significant difference in the average saccade amplitude of high- and low-load trials, the classifier was able to classify trials based on this information significantly better than chance at both the mean saccade and single saccade levels. This implies that there is some structure in the distribution of saccade amplitudes that distinguishes the conditions, that is not being captured by their respective means. Finally, we trained a model to classify the load condition from both the mean fixation duration and saccade amplitude on each trial. This model did not perform significantly better than the model trained on fixation durations alone, *t*(19) = 1.57, *p* = 0.134, *Cohen’s D* = 0.14.

**Table 2:** Mean and SD (in parentheses) of prediction performance for SVMs trained to predict load condition from different gaze metrics in Experiment 1

	Mean fixation duration	Single fixation duration	Mean saccade amplitude	Single saccade amplitude	Mean fixation and saccade
Accuracy	0.73 (0.06)***	0.58 (0.04)***	0.55 (0.05)***	0.53 (0.02)***	0.74 (0.05)***
Precision	0.76 (0.06)	0.66 (0.06)	0.55 (0.05)	0.52 (0.01)	0.76 (0.05)
Recall	0.70 (0.08)	0.30 (0.08)	0.61 (0.12)	0.84 (0.03)	0.72 (0.07)
F1	0.73 (0.07)	0.42 (0.08)	0.57 (0.07)	0.64 (0.01)	0.73 (0.06)

Accuracy was statistically compared to chance level (0.50) with one-sample *t* tests, ***p < 0.001. Precision, recall, and F1 are presented for descriptive purposes, but were not statistically assessed

#### Tone responses

Tones were detected in 98% of tone-present trials in both the low-load and high-load conditions, thus confirming that our use of clearly audible tones was successful in ensuring the tones were perceived. High perceptual load search significantly slowed responses to the tones (*M* = 587 ms) relative to search under low perceptual load (*M* = 549 ms), *t*(19) = 4.60, *p* < 0.001, *Cohen’s D* = 0.34, thus generalising the cross-modal effect of visual perceptual load on processing supra-threshold auditory stimuli (e.g. Molloy et al., 2019) to our new task.

#### Tone effects on fixation durations

**Fig. 2:**
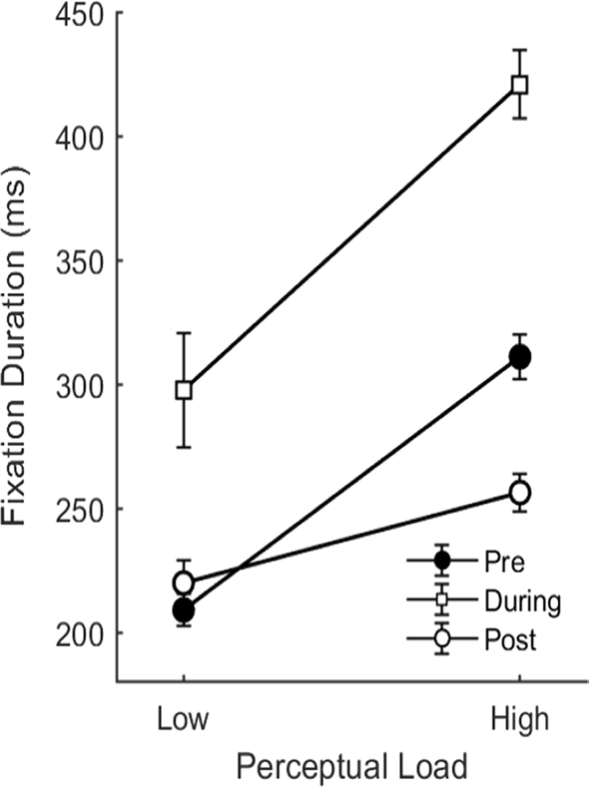
Fixation durations in Experiment 1 as a function of perceptual load and the time of fixation relative to tone presentation (pre-tone, during-tone, and post-tone). Note*.* Error bars represent within participants SEM (Cousineau, 2005; Morey, 2008)

Finally, we also examined the effects of tone presentation on the properties of the gaze. We would expect that following correctly detected tones, any effect of perceptual load should begin to diminish as participants were instructed to abort their search following their tone response. Indeed, when examining the duration of fixations on non-target items this is exactly what we found. A 3 × 2 repeated-measures ANOVA on participants’ fixation durations with the factors tone time (pre-tone, during-tone, post-tone) and perceptual load (low, high) revealed a significant main effect of tone time, *F*(1.43,27.07) = 60.15, *p* < 0.001, *η*^2^ = 0.37, such that fixations during which the tone occurred were of a significantly longer duration (*M* = 359 ms, from an average *n* = 30.4 fixations across the experiment, after target fixations were removed) than fixations prior to the tone (*M* = 260 ms, *n* = 124.1 fixations), *t*(19) = 8.43, *p* < 0.001, *Cohen’s D* = 1.89, or fixations following the tone (*M* = 238 ms, *n* = 31.9 fixations), *t*(19) = 10.29, *p* < 0.001, *Cohen’s D* = 2.30. There was a non-significant trend towards shorter post-tone fixations compared to pre-tone fixations,* t*(19) = 1.86, *p* = 0.070, *Cohen’s D* = 0.42. These main effects were qualified by a significant time × load interaction, *F*(1.23,23.33) = 5.92, *p* = 0.018, *η*^2^ = 0.05 (Fig. 2). This interaction reflected a reduction of the load effect on the duration of post-tone fixations. This was confirmed in follow-up paired comparisons that revealed that while perceptual load was associated with a significant increase in fixation duration in all time conditions; pre-tone: *t*(19) = 11.29, *p* < 0.001, *Cohen’s D* = 2.52; during-tone: *t*(19) = 4.12, *p* < 0.001, *Cohen’s D* = 0.92; post-tone: *t*(19) = 3.64, *p* = 0.002, *Cohen’s D* = 0.81, this effect was significantly smaller in the post-tone fixations (load effect *M* = 36 ms) compared to pre-tone fixations (load effect *M* = 102 ms), *t*(19) = 4.75, *p* < 0.001, *Cohen’s D* = 1.06, and during-tone fixations (load effect *M* = 123 ms), *t*(19) = 2.58, *p* = 0.019, *Cohen’s D* = 0.58. The load effect did not differ between the pre-tone and during-tone fixations, *t*(19) = − 0.78, *p* = 0.448, *Cohen’s D* = − 0.17. Thus, the effect of perceptual load on fixation durations begins to reduce following the tone that indicates that search should be terminated; however, subjects evidently could not disengage from the task immediately.

The results of Experiment 1 establish the effects of perceptual load in a multi-target visual task on participants’ ability to respond to an auditory tone. Despite the visual task involving only gaze-based search with no manual responses, the manual response to an auditory tone was significantly slower when the visual search was more demanding. In addition, the results established a clear marker of perceptual load in the eye gaze data. High perceptual load increased the number of non-target fixations and their durations.

## Experiment 2

Experiment 1 had only a small number of tones that were presented during a saccade (low load *M* = 5.90, high load *M* = 6.75). This prevented us from examining the impact of the tone on saccades that were underway when the tone occurred, and also precluded any strong conclusions regarding the impact of perceptual load on responses to a tone that is presented during a saccade. However, the question of whether perceptual load affects responses to a warning signal applies to periods of saccade equally as much as to periods of fixation. This question is relevant to the design of systems to predict the perceptual load experienced by a user, and by extension, to predict any likely performance impairments. For example, if the effects of perceptual load are not as robust during saccades, systems could be designed to deliver alerts during saccades so as to maximise responses to the warning signals even when the observer is detected to be performing under conditions of high perceptual load. To allow us to examine responses to tones that occur during saccades with increased power, in Experiment 2 we yoked the occurrence of tones to the gaze behaviour of interest. That is, on half the trials tones were only initiated during a fixation, while on the other half of trials, they were initiated upon the detection of a saccade.

### Methods

#### Participants

Twenty-two new participants took part in Experiment 2 (14 females, mean age 22.41 years, SD = 4.06 years). Participants were recruited from the UCL Institute of Cognitive Neuroscience subject mailing list and were compensated for their time at a rate of £7.50 per hour. Participants had normal hearing and normal vision (no glasses or contact lenses) with no self-reported colour vision deficiency or astigmatism. The experiment was approved by the UCL Research Ethics Committee.

#### Apparatus and stimuli

The apparatus was identical to Experiment 1. The stimuli were identical to Experiment 1 with the following exceptions: In order to drive more non-target fixations and saccades, the number of targets in each display was reduced to four (two of each target type; the mapping of stimuli to conditions was unchanged from Experiment 1) and the number of non-targets in each display was increased to 16 (eight of each non-target type).

#### Procedure

The procedure was identical to Experiment 1 with the following exceptions: to correspond with the reduced number of targets, trials were shortened to a maximum of 3.5 s. We added a control such that 50% of tones began during a fixation, and 50% of tones began during a saccade (tones still occurred on 50% of trials overall). Tones on fixation-tone trials and saccade-tone trials were controlled to occur during the first fixation/saccade that occurred after a minimum period of time had elapsed. The minimum periods of time used in each block were 9 equally spaced times between 0.5 and 2.5 s into the trial, presented in random order separately for fixation- and saccade-tone trials. Tone duration was shortened to 30 ms.

#### Eye-tracking parameters

The eye-tracking parameters were identical to those used in Experiment 1.

### Results and discussion

#### Search task

The results of Experiment 2 (Table 3) closely mirrored those of Experiment 1. High perceptual load was associated with a smaller number of targets found (*M* = 1.58) compared to low load (*M* = 2.40), *t*(21) = 21.27, *p* < 0.001, *Cohen’s D* = 4.55, and a greater number of non-target fixations (*M* = 4.24 in the high-load condition, versus *M* = 3.01 in the low-load condition), *t*(21) = 9.33, *p* < 0.001, *Cohen’s D* = 2.27. Average values for each of the assessed gaze properties are presented in Table 2. Again, non-target fixation durations were significantly longer under high load than under low load, *t*(21) = 12.19, *p* < 0.001, *Cohen’s D* = 2.10, and this result was robust to the removal of false positives, *t*(21) = 11.25, *p* < 0.001, *Cohen’s D* = 1.87. In contrast to Experiment 1’s results, saccade amplitudes were shorter under high load than under low load, *t*(21) = 7.36, *p* < 0.001, *Cohen’s D* = 0.94. This is likely to be due to the larger number of non-target items that were inspected in the high-load compared to low-load condition.

**Table 3:** Means and standard deviations (in parentheses) for the gaze metrics as a function of perceptual load in Experiment 2

	Fixation duration (ms)	Fixation duration (ms) excl. False Pos	False positives (# per trial)	Saccade amplitude (degree)
Low load	195 (24)	186 (21)	0.05 (0.03)	5.43 (0.60)
High load	254 (32)	221 (17)	0.23 (0.11)	4.91 (0.52)

#### SVM predictions

Again, an SVM classifier trained on participants’ average fixation durations from each trial was able to classify the load condition of each trial well above chance (Table 4). This pattern of results persisted when classifying the load condition associated with single fixations, but with reduced accuracy. As in Experiment 1, here the classifier was able to classify trials based on saccade amplitudes significantly better than chance at both the mean saccade and single saccade levels. Interestingly, and consistent with the results described above, a model trained to classify load condition from the mean fixation duration and saccade amplitude on each trial this time performed significantly better than the model trained on fixation durations alone, *t*(21) = 3.56, *p* = 0.002, *Cohen’s D* = 0.35.

**Table 4:** Mean and SD (in parentheses) of prediction performance for SVMs trained to predict load condition from different gaze metrics in Experiment 2

	Mean fixation duration	Single fixation duration	Mean saccade amplitude	Single saccade amplitude	Mean fixation and saccade
Accuracy	0.67 (0.06)***	0.58 (0.04)***	0.59 (0.07)***	0.53 (0.02)***	0.69 (0.07)***
Precision	0.72 (0.06)	0.67 (0.06)	0.58 (0.06)	0.52 (0.01)	0.73 (0.07)
Recall	0.58 (0.13)	0.32 (0.08)	0.68 (0.15)	0.83 (0.03)	0.65 (0.12)
F1	0.64 (0.10)	0.42 (0.08)	0.62 (0.09)	0.63 (0.01)	0.68 (0.09)

Accuracy was statistically compared to chance level (0.50) with one-sample *t* tests, *** *p* < 0.001. Precision, recall, and F1 are presented for descriptive purposes, but were not statistically assessed

#### Tone responses

**Fig. 3:**
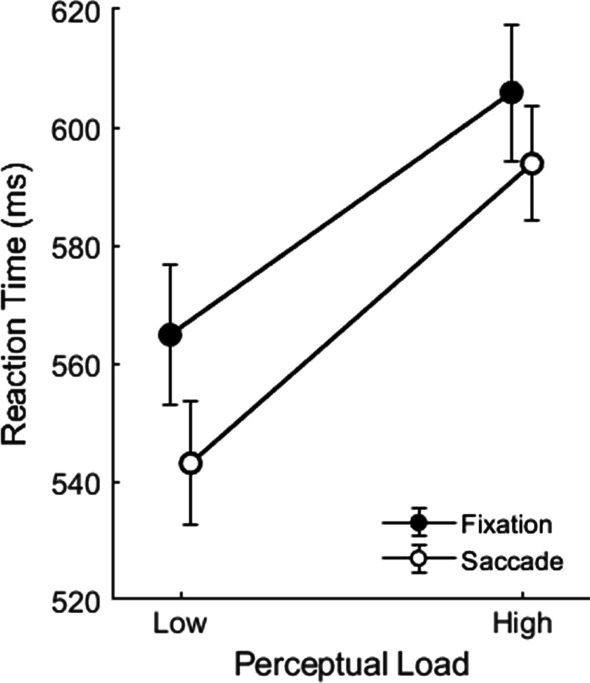
Tone RTs in Experiment 2, as a function of perceptual load and gaze behaviour (tone presented during fixation vs. during saccade). Note. Error bars represent within participants SEM (Cousineau, 2005; Morey, 2008)

Tone detection rates were higher for low-load search (95%) than for high-load search (92%), *t*(21) = 4.07, *p* < 0.001, *Cohen’s D* = 0.54. A 2 × 2 repeated-measures ANOVA on participants tone RTs with the factors perceptual load (low, high) and tone time (during fixation, during saccade) revealed a significant main effect of perceptual load, *F*(1,21) = 7.95, *p* = 0.010, *η*^2^ = 0.28, such that tone responses were significantly slower in the high-load compared to the low-load conditions (Fig. 3), as in Experiment 1. The main effect of tone time approached significance, *F*(1,21) = 4.23, *p* = 0.052, *η*^2^ = 0.17, with a non-significant trend towards faster responses to the tone when it occurred during a saccade. There was no significant interaction between perceptual load and tone time, *F*(1, 21) = 1.00, *p* = 0.329, *η*^2^ = 0.05. As can be seen in Fig. 3, tone responses were similarly slowed by perceptual load, irrespective of whether the tone was presented during a fixation or a saccade.

#### Tone effects on fixation durations

**Fig. 4:**
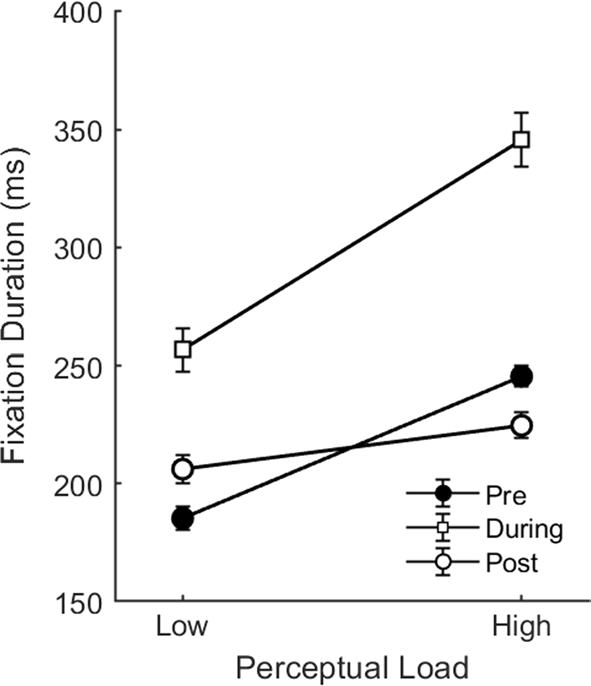
Fixation durations in Experiment 2 as a function of perceptual load and the time of fixation relative to tone presentation (pre-tone, during-tone, and post-tone). Note. Error bars represent within participants SEM (Cousineau, 2005; Morey, 2008)

A 3 × 2 repeated-measures ANOVA on participants’ fixation durations with the factors Time (pre- tone, during-tone, post-tone) and perceptual load (low, high) revealed a significant main effect of perceptual load, *F*(1,21) = 58.03, *p* < 0.001, *η*^2^ = 0.22, such that high-load search produced significantly longer fixations than low-load search, as established previously. There was also a significant main effect of Time, *F*(1.57,33) = 91.33, *p* < 0.001, *η*^2^ = 0.46, such that fixation durations were significantly longer for the fixation during which the tone occurred (*M* = 301 ms, from average *n* = 38.3 fixations) than for fixations prior to the tone (*M* = 215 ms, *n* = 162.5 fixations), *t*(21) = 10.54, *p* < 0.001, *Cohen’s D* = 2.25, or fixations following the tone (*M* = 215 ms, *n* = 71.6 fixations), *t*(21) = 10.31* p* < 0.001, *Cohen’s D* = 2.20. Post-tone fixations did not differ from pre-tone fixations (*t* < 1). These main effects were qualified by a significant Time × Load interaction, *F*(1.36,28.45) = 15.96, *p* < 0.001, *η*^2^ = 0.06 (Fig. 4). As can be seen in the figure, similarly to Experiment 1, this interaction also reflected a smaller effect of load on post-tone fixations compared to pre-tone and during-tone fixations. This was confirmed in follow-up paired comparisons showing that while the load effect was present in fixation durations in all time conditions; pre: *t*(21) = 12.85, *p* < 0.001, *Cohen’s D* = 2.74; during: *t*(21) = 5.70, *p* < 0.001, *Cohen’s D* = 1.22; post: *t*(21) = 2.53, *p* = 0.019, *Cohen’s D* = 0.54, it was smaller in the post-tone fixations (load effect *M* = 11 ms) compared to pre-tone fixations (load effect *M* = 60 ms), *t*(21) = 8.72, *p* < 0.001, *Cohen’s D* = 1.86, and during-tone fixations (load effect *M* = 89 ms), *t*(21) = 4.77, *p* < 0.001, *Cohen’s D* = 1.02. During-tone fixations showed a trend for a larger load effect than that found for pre-tone fixations, but this was not significant, *t*(21) = 1.90, *p* = 0.071, *Cohen’s D* = 0.41.

#### Tone effects on saccade amplitude

**Fig. 5:**
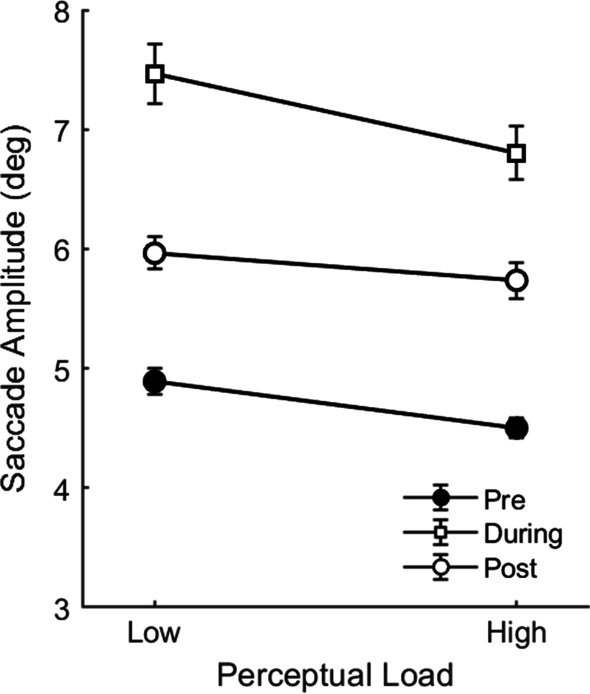
Saccade amplitudes in Experiment 2 as a function of perceptual load and the time of fixation relative to tone presentation (pre-tone, during-tone, and post-tone). *Note.* Error bars represent within participants SEM (Cousineau, 2005; Morey, 2008)

In Experiment 2, we also examined the effects of tone presentation on saccade amplitudes. A 3 × 2 repeated-measures ANOVA on participants’ saccade amplitudes with the factors Time (pre- tone, during-tone, post-tone) and perceptual load (low, high) revealed a significant main effect of perceptual load, *F*(1,21) = 11.24, *p* = 0.003, *η*^2^ = 0.03, such that high-load search produced significantly shorter saccades than low-load search, as reported earlier. There was also a significant main effect of Time, *F*(1.34,28.03) = 77.38, *p* < 0.001, η^2^ = 0.64, such that saccade amplitudes were significantly longer for the saccade during which the tone occurred (*M* = 7.13°, from average *n* = 63.8 saccades) than for saccades prior to the tone (*M* = 4.70°, *n* = 194.1 saccades), *t*(21) = 11.24, *p* < 0.001, *Cohen’s D* = 2.40, or saccades following the tone (*M* = 5.85°, *n* = 153.5 saccades), *t*(21) = 5.41* p* < 0.001, *Cohen’s D* = 1.15. Post-tone saccade amplitudes were significantly shorter than those of pre-tone saccades, *t*(21) = 10.60, *p* < 0.001, *Cohen’s D* = 2.26. There was no significant Time × Load interaction; however, *F*(1.40,29.39) = 1.13, *p* = 0.317, *η*^2^ < 0.01 (Fig. 5).

Experiment 2 thus replicated the main results of Experiment 1 that search for high-perceptual-load targets in multi-target search slowed detection of cross-modal target stimuli, and increased fixation durations. It also confirmed that the effects of perceptual load on tone detection are continuous throughout the search, rather than being limited to times of fixation. Finally, this experiment demonstrated that at least under some circumstances (e.g. with a smaller number of targets, and a greater number of tones presented during saccades) saccade amplitudes can also distinguish between high- and low-load search.

## General discussion

We sought to determine the gaze correlates of increased perceptual load during multi-target visual search. Participants searched for multiple visual targets under low- (feature targets) or high-load (conjunction targets) search conditions and responded to auditory detection targets. The results showed that increased perceptual load in the visual task resulted in fewer targets found and slowed responses to the auditory stimulus. Crucially, in both experiments, the level of perceptual load was also distinguished by consistent changes in the duration of eye fixations, with high-load search producing a significant extension to the average fixation duration relative to low-load search. This pattern of extended fixations under high load was consistent throughout the experiment and the trial (excepting the first and last 500 ms; see Additional file 1), thus providing a stable indicator of perceptual load. By having participants complete a search task in which the stimuli were perceptually matched and including an independent verification of perceptual load (the auditory detection stimuli), we were able to show conclusively that these modulations of gaze properties were due to differences in the attentional requirements of the task. These results add to a growing body of studies that extend the effects of perceptual load on awareness to different sensory modalities than the one being attended. Here we show that perceptual load in a visual search task requiring no overt response (apart from lingering the gaze on targets) still results in slowed manual responses to an auditory signal.

In safety–critical industries, the effects of high perceptual load could result in a user failing to notice or respond to a warning signal. Developing systems to predict perceptual load in these scenarios could be critically important, because it allows automated systems to compensate for expected perceptual deficits when warning the human user that they must switch attention to a different task (e.g. back from their non-driving task to driving in Level 3 automation; SAE, 2021). In particular, both the reduced awareness of the world outside the focus of attention that occurs under high perceptual load (e.g. Cartwright-Finch & Lavie, 2007; Macdonald & Lavie, 2008) and the reduced ability to orient to an exogenous cue (e.g. Santangelo et al., 2008) are known to extend cross-modally, resulting in a reduced ability to detect or orient towards sounds and tactile stimuli in conditions of high visual perceptual load (e.g. Macdonald & Lavie, 2011; Murphy & Dalton, 2016; Raveh & Lavie, 2015; Santangelo et al., 2007; Santangelo & Spence, 2007; see also Molloy et al., 2015, 2019 for demonstrations of the reduced neural signal to sound in conditions of high visual load). In cases of high levels of automation (Level 3 automation), drivers are free to engage in non-driving tasks such as internet browsing; however, they may be required to take over control of the vehicle under some scenarios. If these tasks involve a higher level of perceptual load, this may lead to safety–critical consequences if the driver cannot detect, or responds too slowly to, a TOR (or indeed to any signal indicating system failure). However, if automation is able to detect such a state from the impact of perceptual load on the driver’s pattern of gaze behaviour, the AI system can take action to compensate for the user’s reduced attention (e.g. present the TOR earlier to accommodate for slower responses). Our results suggest that systems designed to measure the duration of fixations while users perform non-driving tasks may be useful for this purpose. Our machine learning analysis was able to predict the load condition above chance levels from fixation duration or saccade amplitude in both experiments with a small increase in accuracy from combining both measures in Experiment 2. This was achieved with no model tuning and with a relatively small training dataset. With larger datasets, purpose-engineered models, and additional predictor information (e.g. body posture, task information, etc.) in-car attention-prediction systems should be able to achieve high levels of accuracy.

Of particular importance for application of the current results are the findings that, apart from the very start and end of a trial, there was little effect of within-trial time on fixation durations (only a very small effect in the low-load condition of Experiment 2; see Additional file 1). On the surface, these results seem to conflict with those described earlier from Mills et al. (2011) who found fixation durations increased across within-trial time in four separate tasks (free viewing, viewing for memory, viewing for pleasantness rating, and search). However, it is worth noting that in the free-viewing and search conditions of Mills et al. (2011), the pattern of results also showed a rapid increase in fixation duration at the start of the trial followed by a relative plateau in which fixation durations changed very little throughout the rest of the trial. Thus, in the conditions most analogous to ours, their results were quite similar. The pattern of steady fixation durations across time is important for applicability, as it shows that a single model of fixation duration can be sufficient to detect differences in load, without needing to account for the duration of time on task, provided the earliest segment of data is excluded.

Supporting our claim that the effects of load on fixation durations were due to the attentional requirements of the task, we found that following the tone, after the task had switched from visual fixations to a manual tone response, the effect of perceptual load on fixation durations was drastically reduced but not eliminated. This is an interesting result, as on the one hand it reaffirms that the effects of perceptual load indicate the level of attentional engagement in the task (being strongest while a person is fully engaged in the task), yet on the other hand it also demonstrates that the effects of perceptual load do not disappear immediately upon the requirement to switch tasks. The effect of perceptual load seems to persist for some period, consistent with both the slowed responses to the tone in the high-load condition, and with the increased duration of fixations measured in the tone response period. Future studies could use a post-switch task with a longer measurement period to allow the time-course of load-effect extinction to be characterised. Examination of this time-course could provide important indications of the likely neural underpinnings of perceptual load, and, in applied contexts, the minimum amount of time for a user to regain full focus on the post-switch task.

Our findings that higher perceptual load substantially increased fixation duration even when the visual properties of the stimuli were matched between conditions, are consistent with past results suggesting fixation duration in visual search is related to target–non-target similarity (Becker, 2011; Horstmann et al., 2016; Reingold & Glaholt, 2014; Shen et al., 2003). Manipulation of target–non-target similarity has previously been demonstrated to be a robust manipulation of perceptual load resulting in reduced processing of search-irrelevant stimuli (e.g. an irrelevant distractor presented in the periphery) in conditions of high load (e.g. Lavie & Cox, 1997; Roper et al., 2013). However, past studies have typically measured the effects of target–non-target similarity on gaze behaviour in displays that have a different appearance between the different conditions. This has two major limitations. The first is that any effects on fixation duration could be attributed to a greater demand on visual acuity (which requires, therefore, longer foveation), rather than perceptual load itself. Acuity manipulations increase difficulty by limiting the amount of information available to the visual system, rather than by taxing attention. The second limitation is with respect to the applied utility of the gaze measure of load. Displays that differ between conditions of perceptual load do not require a measure of gaze pattern to detect the increased load, since this could be detected on the basis of the difference in visual stimuli themselves. Indeed, Nagle and Lavie (2020) recently demonstrated that a high level of model prediction can be achieved to predict participants’ perceived complexity (a proxy for load) of a large variety of natural images from the properties of the images themselves.

We note that while our feature (shape) versus conjunctions (shape and colour) design allowed us to use the same displays between conditions of differing levels of perceptual load, this manipulation necessarily involves an increase in visual short term (VSTM) load in the high-load condition as participants are required to remember more target features. This increase may have contributed to the perceptual load effects seen in the present results, especially since much research has demonstrated that VSTM maintenance draws on similar neural representation resources to those involved in perception (e.g. Pasternak & Greenlee, 2005). Indeed, VSTM load produces similar effects to those of perceptual load on various measures ranging from neural responses related to the detection of a secondary task stimulus (Konstantinou et al., 2012), to behavioural measures of the effects of load on perception through detection sensitivity, contrast response function, and distractor interference effects (Konstantinou & Lavie, 2013, 2020; Konstantinou et al., 2014). It is important to note, however, that VSTM load is not a necessary component for producing the effects of perceptual load. Load effects have been shown resulting from many manipulations that do not include increased memory load, such as the aforementioned manipulations of search set size or target–non-target similarity. Each of these manipulations has also been found to affect detection and reduce neural responses to an unrelated visual or auditory stimulus, similarly to the performance cost we establish here (e.g. Macdonald & Lavie, 2008, 2011; Raveh & Lavie, 2015; Molloy et al., 2015). Moreover, other manipulations of working memory load that do not draw on shared task-relevant resources (e.g. verbal working memory load during a visual task) result in the opposite effects to both perceptual load and VSTM load. That is, increased cognitive memory load results in increased distraction, increased neural responses, and increased visual detection sensitivity to a task-unrelated stimulus (e.g. de Fockert et al., 2001; Lavie, 2000; Konstantinou et al., 2014; Konstantinou & Lavie, 2013). These effects are attributed to reduced cognitive control over task processing priorities with increased cognitive working memory load. An interesting question for future research would therefore be to compare the impact of cognitive control load (e.g. verbal working memory load) with VSTM load and perceptual load on fixation durations and saccade amplitudes during the visual search task reported here. This question is particularly interesting since pupil measures appear insensitive to whether perceptual load or working memory load is varied. In contrast to what is found for behavioural and neural measures, both forms of load result in increased pupil dilation (Beatty & Kahneman, 1966; Kahneman & Beatty, 1966; Porter et al., 2010).

Another critical difference between the present study design and previous work examining gaze properties related to search load is that past studies have typically involved search for one target only. In the present study, we have extended our understanding of the effect of perceptual load on the gaze to multi-target search, which is more akin to real-world tasks such as internet use. Another situation in which multi-target search has been examined in relation to gaze behaviour is in the study of visual foraging (e.g. Jóhannesson et al., 2016; Tagu & Kristjánsson, 2022; Wolfe, 2020). In foraging studies, participants are required to perform an exhaustive search of all targets within a given many-item display, and search for either targets distinguished by a single feature (low-load search) or a conjunction of features (relatively more high-load search). Unlike the present results, and studies reviewed earlier, these studies have shown a *reduction* of fixation duration when conjunction search is performed. However, several critical differences between our task design and the tasks designed to examine visual foraging preclude a direct comparison of the results between the different tasks. Specifically, our displays of 20 items in total were far less crowded compared to the dense displays that are typically employed in foraging tasks (e.g. 80 items were used in Tagu & Kristjánsson, 2022). Moreover, our tasks permitted non-target inspection, since many real-world search tasks involve careful inspections of non-target items before these are deemed not to be the target. In contrast, Tagu and Kristjánsson (2022) penalised participants for fixating distractors for more than 350 ms by having them begin the full 80-item search over again. With their combination of dense displays and the penalty on lingering fixation on non-target items, at least some of the shorter fixation durations in their high-load condition may have been attributed to a bias of participants shortening their fixations to avoid the large penalty of starting the search over again if they accidentally fixated a non-target for too long. Finally, we designed our task to avoid a simplifying strategy of long runs, which is more likely in higher load conditions, as shown in prior foraging studies (e.g. Jóhannesson et al., 2016; Kristjánsson et al., 2014). Such a simplifying strategy may reduce the engagement of attentional resources (Kristjánsson et al., 2018). The ‘load-induced blindness or deafness’ that is of most concern for safety–critical situations may be less likely to occur in high-load conditions of tasks that lend themselves to a simplifying, load-reducing strategy. Thus, our high-load search required focusing on each item in the high-load condition to determine the specific colour by orientation combination within that item.

In the present study, we found an effect of perceptual load on saccade amplitudes (which were longer in low load than in high load) in Experiment 2, but no such relationship was observed in Experiment 1. The reason for this is most likely due to the differences between experiments in the number of targets and therefore their proximity to each other. In Experiment 1, 50% of the search items were targets, so the distance the eyes needed to move to find the next potential target was shorter. By contrast, in Experiment 2 where only 20% of the items were targets, the eyes had further to travel to land on prospective targets. It is likely that in Experiment 2, nearby distractors could be rejected, and distant targets recognised, under low-load, whereas this capacity was reduced in high-load search, giving rise to shorter saccades in the high-load condition to inspect nearby items. This explanation is consistent with the idea of perceptual load reducing the size of the attentional window (Cave & Chen, 2016; Hulleman & Olivers, 2017). Thus, saccade metrics may differentiate perceptual load under certain conditions, but they are highly sensitive to the actual distance between the relevant items in a search, and this can change independently of perceptual load. Clearly then, fixation durations provide a more reliable and generalizable indicator of perceptual load. Future work could perform a systematic manipulation of set size with this paradigm to determine the boundary conditions for this saccade effect.

Past studies have found that the frequency of secondary target occurrence does not modulate the impact of perceptual load on secondary target reports. For example, in Raveh and Lavie (2015) there was no difference in the influence of load on a secondary auditory detection task between an experiment in which the target was presented on 17% of trials, and an experiment in which it was present on 50% of trials and a present/absent response was made on every trial. In the present experiments, auditory detection targets were present on 50% of trials but were infrequent relative to the multiple primary visual task items present on each trial. The relative frequency of tone to visual task items was somewhat higher in Experiment 2 since our trials were shorter compared to Experiment 1. Yet our two experiments showed similar load effects on secondary task reaction time and fixation duration during search, but differences on other measures (secondary target detection, saccade amplitude). It will be an important direction for future work to examine the influence between secondary target frequency and secondary target responses in the current paradigm. In particular, it will be important to examine the influence of load on secondary target detection under conditions in which the secondary target is exceedingly rare and unexpected, as is likely to be the case with a TOR occurrence in highly automated driving.

While we have provided strong evidence for an influence of perceptual load on fixation durations, it is important to note that perceptual load is unlikely to be the only influence on fixation durations during highly automated driving. Other influences on fixation duration will contribute to the overall fixation pattern of the user (e.g. reading content, Kliegl et al., 2004, Degno et al., 2019; specific task, Mills et al., 2011, Nuthmann et al., 2010; current goals, Jang et al., 2021; learning, Harris & Remington, 2020; semantic and syntactic content of the scene, Vo & Henderson, 2009, Coco et al., 2020, etc.), thus a model that predicts user perceptual load from fixation duration will need to account for these factors. Similar multi-factor relationships exist for other possible attention indicators (e.g. pupil diameter). Considerable focus will need to be devoted to examining the interactions of these factors and others on attention indicators in future studies.

In summary, multi-target visual search for conjunctions of features (shape and colour) induces a state of high perceptual load compared to search of the same displays for a single target feature (shape), even when no manual response is required. High perceptual load leads to both poorer search performance and poorer responding to sound stimuli that are not part of the search, generalising a growing body of cross-modal load research across to a novel multi-target gaze-dependent visual search task. Critically, this increase in perceptual load was accompanied by a corresponding increase in the duration of fixations throughout the search. Increased fixation durations with perceptual load could be important indicators for user monitoring and automated systems that interact with humans. They may allow for automated systems to predict potentially safety–critical situations whereby the user may either fail to notice a warning signal due to load-induced blindness or deafness (Macdonald & Lavie, 2008, 2011; Raveh & Lavie, 2015; Molloy et al., 2015; 2019), or they may respond significantly slower when speed of response is essential for user safety.

### Supplementary Information

**Additional file 1.** Analysis of fixation duration and saccade amplitude for each load condition in Experiments 1 and 2, broken down by within-trial time, and time across the experiment.

## Footnotes

1 Current models in commercial use allow the driver to use certain features of the infotainment system that are only enabled during the ‘auto-pilot’ drive mode, such as a Tetris video game.
