# Supplementary material for: Establishing gaze markers of perceptual load during multi-target visual search
Source: Cogn Res Princ Implic. 2023 Aug 31;8:56. doi: 10.1186/s41235-023-00498-7 (PMC10468466; doi:10.1186/s41235-023-00498-7)
Supplement: Supplementary file 1 — Additional file 1. Analysis of fixation duration and saccade amplitude for each load condition in Experiments 1 and 2, broken down by within-trial time, and time across the experiment. [file 41235_2023_498_MOESM1_ESM.docx]

**Supplementary Materials**

Supplementary Analysis 1:

We sought to determine the effect of time both within a trial and across the experiment on fixation duration in Experiment 1. Inspection of the results across within-trial time (**Figure S1A**) indicated a potentially nonlinear fixation duration profile, so we computed separate quadratic mixed effects models for the low- and high-load conditions. These regressions predicted fixation duration from the fixed effects of trial number (for time across the experiment session) and fixation-start-time relative to trial onset (for within-trial time). Participants were included as a random effect. Trials in which a tone occurred were excluded from this analysis.

The quadratic regression for the low-load search condition indicated significant prediction of fixation duration from both the quadratic (*b* = -4.79x10^-6^, *p* < .001) and linear (*b* = .04, *p* < .001) relationships with within-trial time. In high-load search, we observed significant prediction of fixation duration from the quadratic (*b* = -5.77x10^-6^, *p* < .001) and linear (*b* = .04, *p* < .001) relationships with within-trial time. However, as can be seen in **Figure S1A**, these results were largely driven by fixations occurring in the first and last 500 ms of the trial. The shorter fixations in the first 500ms are likely driven by brief exploratory fixations upon the beginning of the trial, before a target is selected for fixation. Similarly, the brief fixations at the end of the trial are likely driven by the majority of late fixations being excluded for not finishing before the trial end, thus leaving only the briefest fixations in this time range. Exclusion of these fixations from the above regressions resulted in no significant quadratic or linear associations with within-trial time in either load condition (all *p*s > .26).

The linear relationship with trial number (time across the experiment) was significant in the low load condition (*b* = .26, *p* = .018), reflecting longer fixation durations as the experiment went on, but the quadratic relationship was not (*b* = -6.22x10^-4^, *p* = .070). In high-load search, trial number was also not a significant quadratic (*b* = 4.86x10^-4^, *p* = .181) or linear (*b* = -.18, *p* = .078) predictor of fixation duration (**Figure S1B**).

**Figure S1.**

*Fixation durations from Experiment 1*


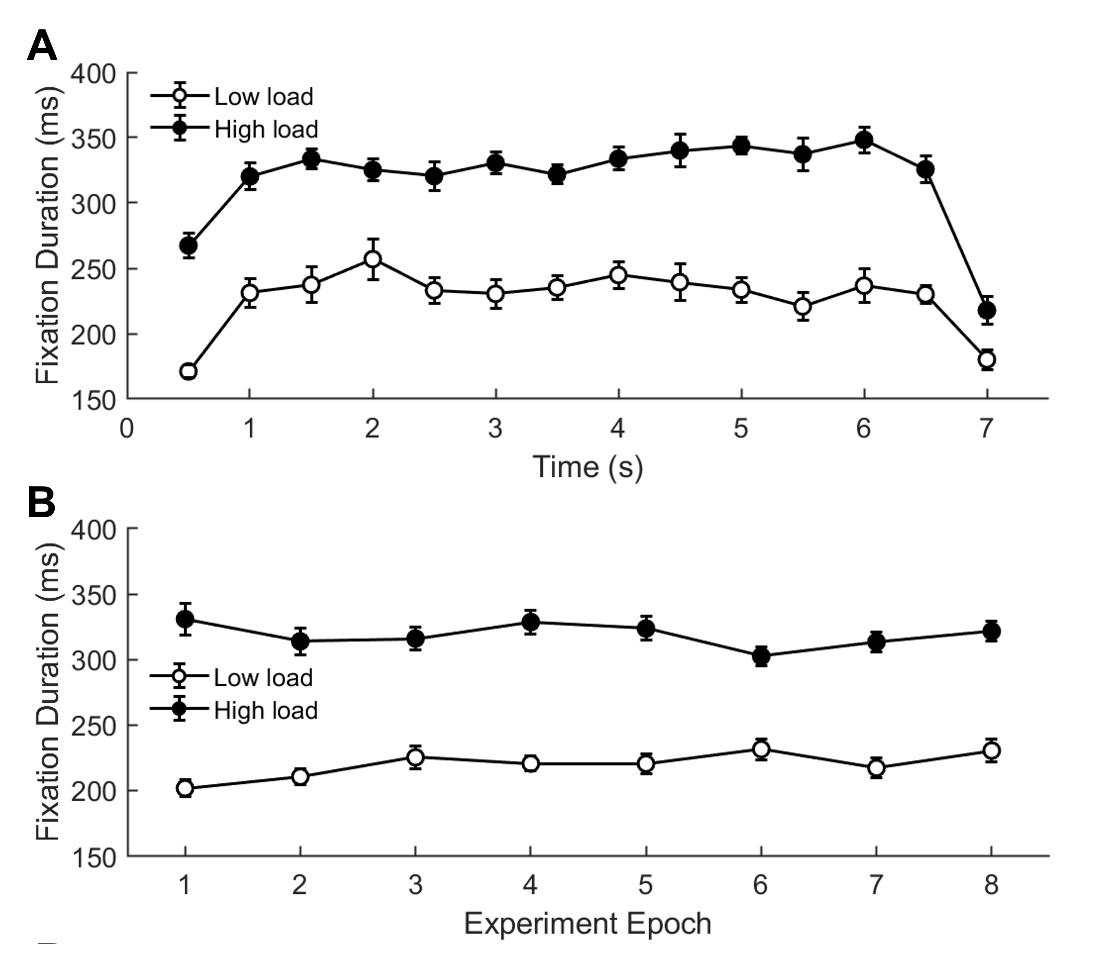


*Note.* A) Fixation duration over within-trial time. Each time-point represents an average of data from the previous 0.5 seconds. B) Fixation duration over the course of the experiment. Experiment epochs represent average data from seven even groups of experimental trials. Error bars represent within-participants SEM (Cousineau, 2005; Morey, 2008). Note, data here are binned for display purposes only. Regression analyses were performed using the continuous fixation start times and trial numbers.

The same analysis performed on saccade amplitudes from the low-load condition showed significant quadratic (*b* = -4.16x10^-8^, *p* < .001) and linear (*b* = 4.82x10^-4^, *p* < .001) associations with within-trial time in Experiment 1 (**Figure S2A**). The results were largely the same in the high-load condition (quadratic: *b* = -4.26x10^-8^, *p* < .001; linear: *b* = 4.26x10^-4^, *p* < .001). There were no associations with trial number in either the low load (quadratic: *b* = 5.22x10^-6^, *p* = .213; linear: *b* = -.001, *p* = .445), or high load conditions (quadratic: *b* = -9.26x10^-7^, *p* = .802; linear: *b* = 6.97x10^-4^, *p* = .495), as shown in **Figure S2B**.

**Figure S2.**

*Saccade amplitudes from Experiment 1.*


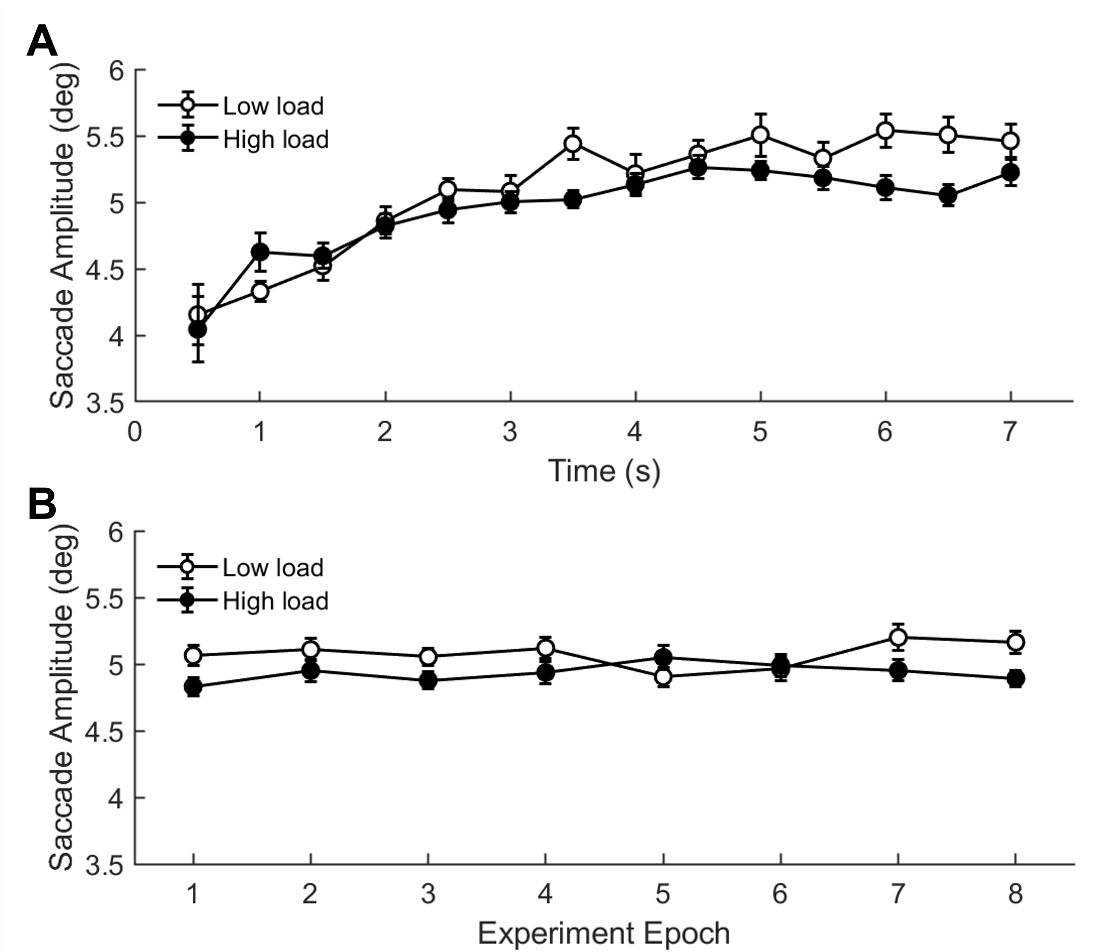


*Note.* A) Saccade amplitudes over within-trial time. Each time-point represents an average of data from the previous 0.5 seconds. B) Saccade amplitude over the course of the experiment. Experiment epochs represent average data from seven even groups of experimental trials. Error bars represent within-participants SEM (Cousineau, 2005; Morey, 2008). Note, data here are binned for display purposes only. The regression analyses were performed using the continuous saccade start times and trial numbers.

Supplementary Analysis 2:

As in Experiment 1, we examined the effects of within- and across-trial time on properties of the gaze in Experiment 2. We first performed quadratic regressions separately for low- and high-load, predicting fixation duration from fixation start time (within-trial time) and trial number (across-trial time). In low-load search, we found significant quadratic (*b* = -1.57x10^-5^, *p* < .001) and linear (*b* = .05, *p* < .001) relationships between fixation duration and within-trial time (**Figure S3A**). The same results were found in the high-load condition (quadratic: *b* = -2.18x10^-5^, *p* < .001; linear: *b* = .06, *p* < .001). Unlike in Experiment 1, exclusion of the first and final 500ms of the trial did not abolish the effects of fixation start time in the low-load condition (quadratic: *b* = -8.27x10^-6^, *p* = .013; linear: *b* = .02, *p* = .041), but they did in the high-load condition (quadratic: *b* = -5.22x10^-6^, *p* = .159; linear: *b* = .007, *p* = .595).

There were no significant relationships between fixation duration and trial number in either the low load (quadratic: *b* = 5.80x10^-4^, *p* = .169; linear: *b* = -.13, *p* = .292) or high load conditions (quadratic: *b* = 5.28x10^-4^, *p* = .131; linear: *b* = -.19, *p* = .064), although the high-load condition did show a non-significant linear trend (**Figure S3B**).

**Figure S3.**

*Fixation durations from Experiment 2*


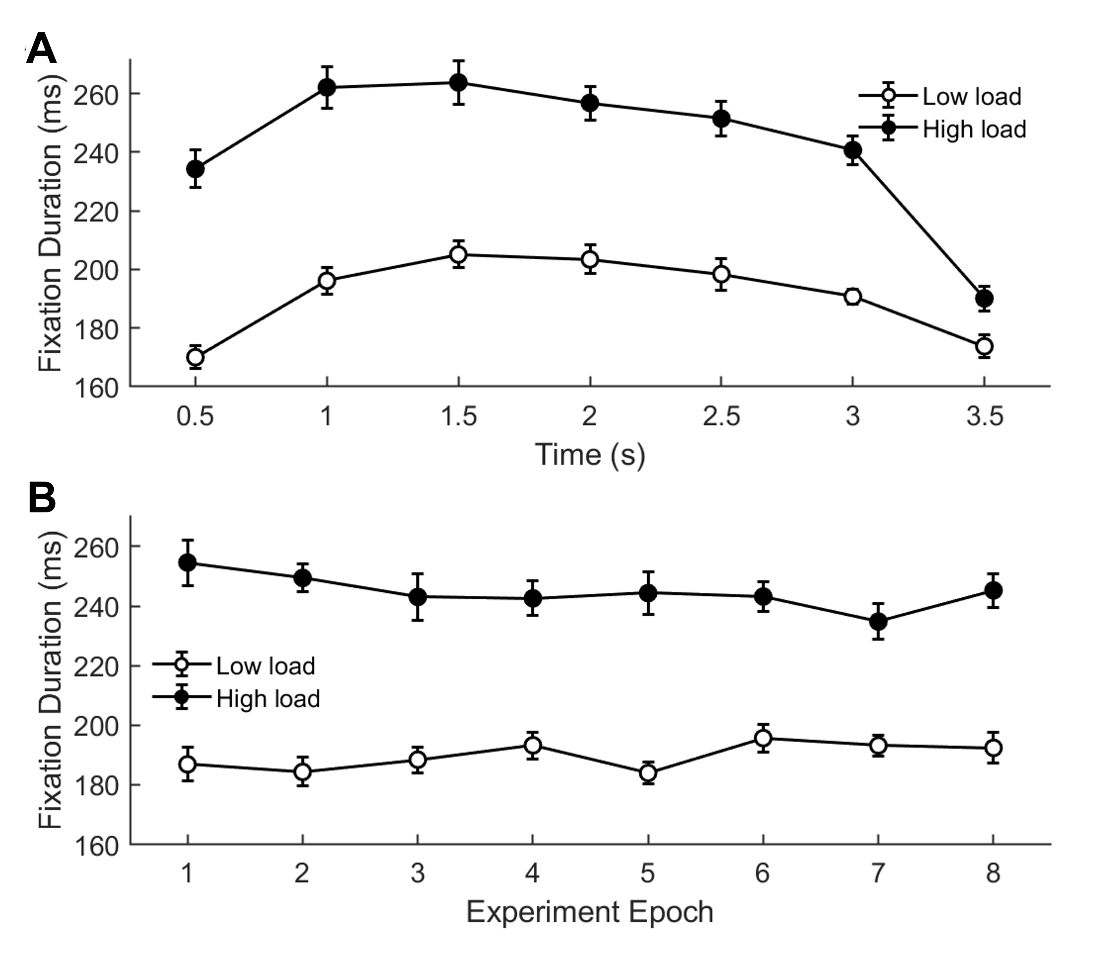


*Note.* A) Fixation duration over within-trial time. Each time-point represents an average of data from the previous 0.5 seconds. B) Fixation duration over the course of the experiment. Experiment epochs represent average data from seven even groups of experimental trials. Error bars represent within-participants SEM (Cousineau, 2005; Morey, 2008). Note, data here are binned for display purposes only. The regression analyses were performed using the continuous fixation start times and trial numbers.

The quadratic regression of saccade amplitude against saccade onset time (within-trial time) and trial number for the low-load condition in Experiment 2 revealed significant quadratic (*b* = -3.24x10^-7^, *p* < .001) and linear (*b* = .002, *p* < .001) effects of within-trial time (**Figure S4A**), and no effects of trial number (quadratic: *b* = -1.52x10^-5^, *p* = .135; linear: *b* = .004, *p* = .223) (**Figure S4B**). The same pattern of results was observed in the high-load condition, with significant quadratic (*b* = -3.44x10^-7^, *p* < .001) and linear (*b* = .002, *p* < .001) associations between saccade-amplitude and saccade onset time, and no relationships with trial number (quadratic: *b* = 3.44x10^-7^, *p* = .952; linear: *b* = -3.63x10^-4^, *p* = .826).

**Figure S4.**

*Saccade amplitudes from Experiment 2.*


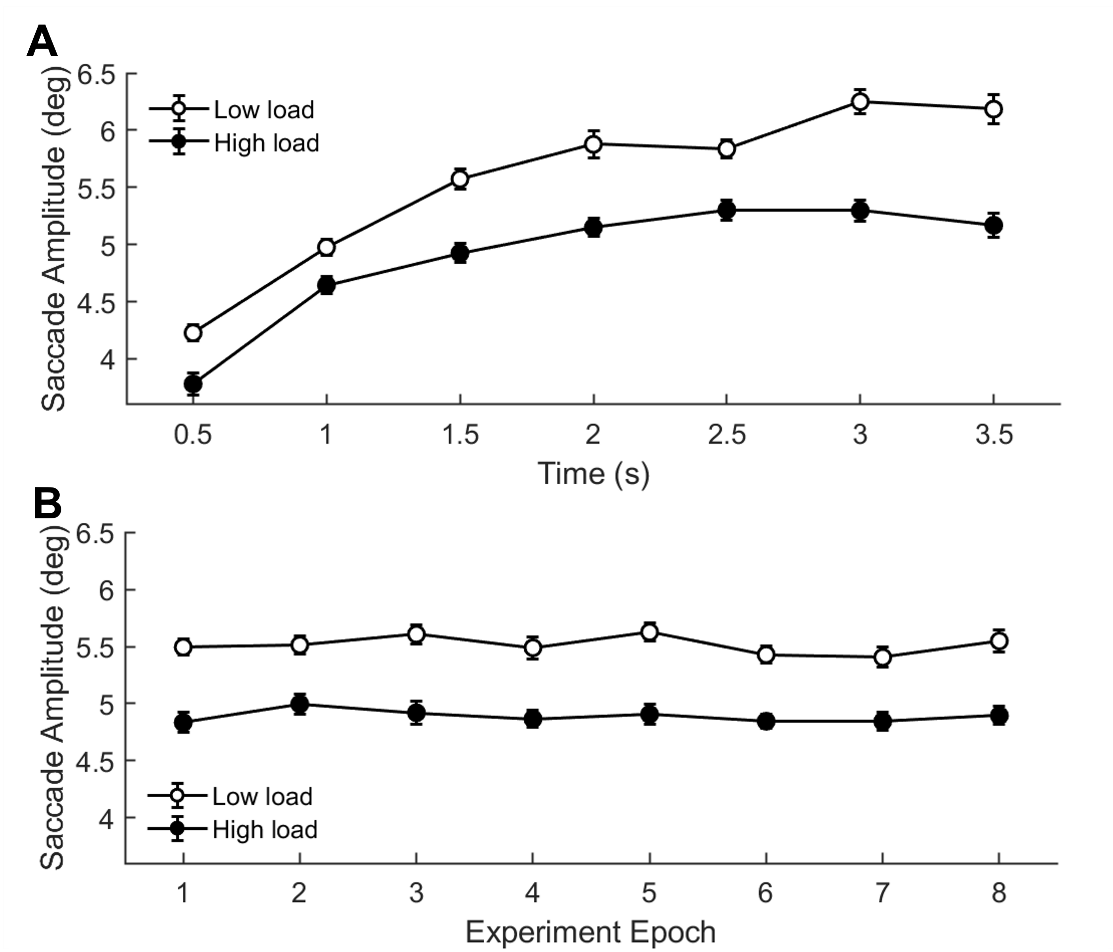


*Note.* A) Saccade amplitudes over within-trial time. Each time-point represents an average of data from the previous 0.5 seconds. B) Saccade amplitude over the course of the experiment. Experiment epochs represent average data from seven even groups of experimental trials. Error bars represent within-participants SEM (Cousineau, 2005; Morey, 2008). Note, data here are binned for display purposes only. The regression analyses were performed using the continuous saccade start times and trial numbers.
